# Supplementary material for: Auxiliary Discrminator Sequence Generative Adversarial Networks for Few Sample Molecule Generation
Source: J Chem Inf Model. 2025 Sep 22;65(19):10311–22. doi: 10.1021/acs.jcim.5c01737 (PMC12529764; doi:10.1021/acs.jcim.5c01737)
Supplement: Supplementary file 1 [file ci5c01737_si_001.pdf]

Auxiliary Discrminator Sequence Generative Adversarial Networks (ADSeqGAN) for Few Sample Molecule Generation

Haocheng Tang,[*,†] Jing Long,[*,‡] Beihong Ji,[*,†] and Junmei, Wang[*,†]

†*School of Pharmacy, University of Pittsburgh, Pittsburgh, Pennsylvania 15261, United States*

‡*School of Software & Microelectronics, Peking University, Beijing, 100871, China*

E-mail: hat170@pitt.edu; jing.long0926@gmail.com; bej22@pitt.edu; juw79@pitt.edu

Supplementary Information

Details of nucleic acids binders and central nervous system drugs

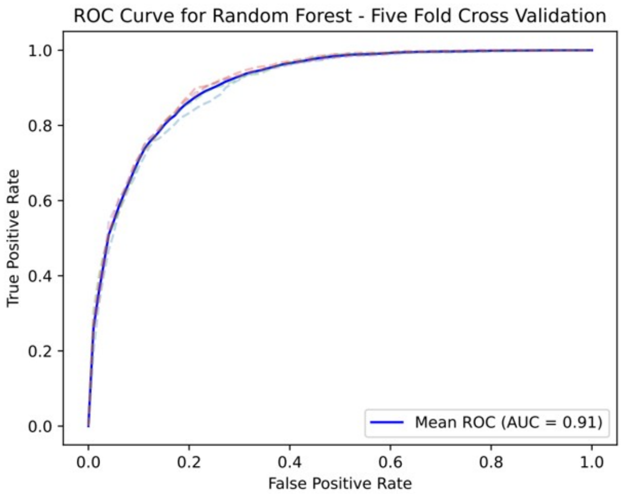

Figure S1: Evaluation of random forest classifier of NA and Pro datasets.

Features to be chosen as auxiliary input: 'NumAromaticRings', 'HallKierAlpha', 'BertzCT', 'PEOE_VSA8', 'VSA_EState6', 'NumAromaticCarbocycles', 'SlogP_VSA6', 'SMR_VSA7', 'MolMR', 'BalabanJ', 'fr_bicyclic', 'MinEStateIndex', 'Chi1', 'FpDensityMorgan1', 'Chi1n', 'Chi0n', 'LabuteASA', 'Ipc'.

Parameters: n_estimators=100, random_state=42.

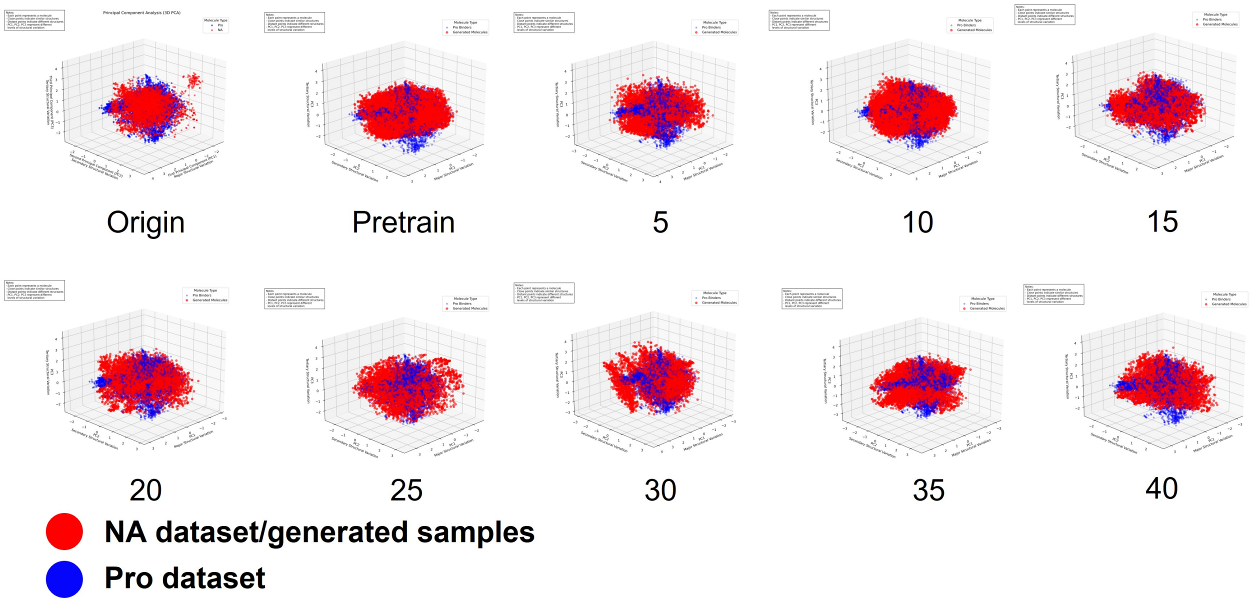

Figure S2: Principal component analysis of the characteristic molecular fingerprint fragments of NA dataset and generated NA samples with original Pro dataset

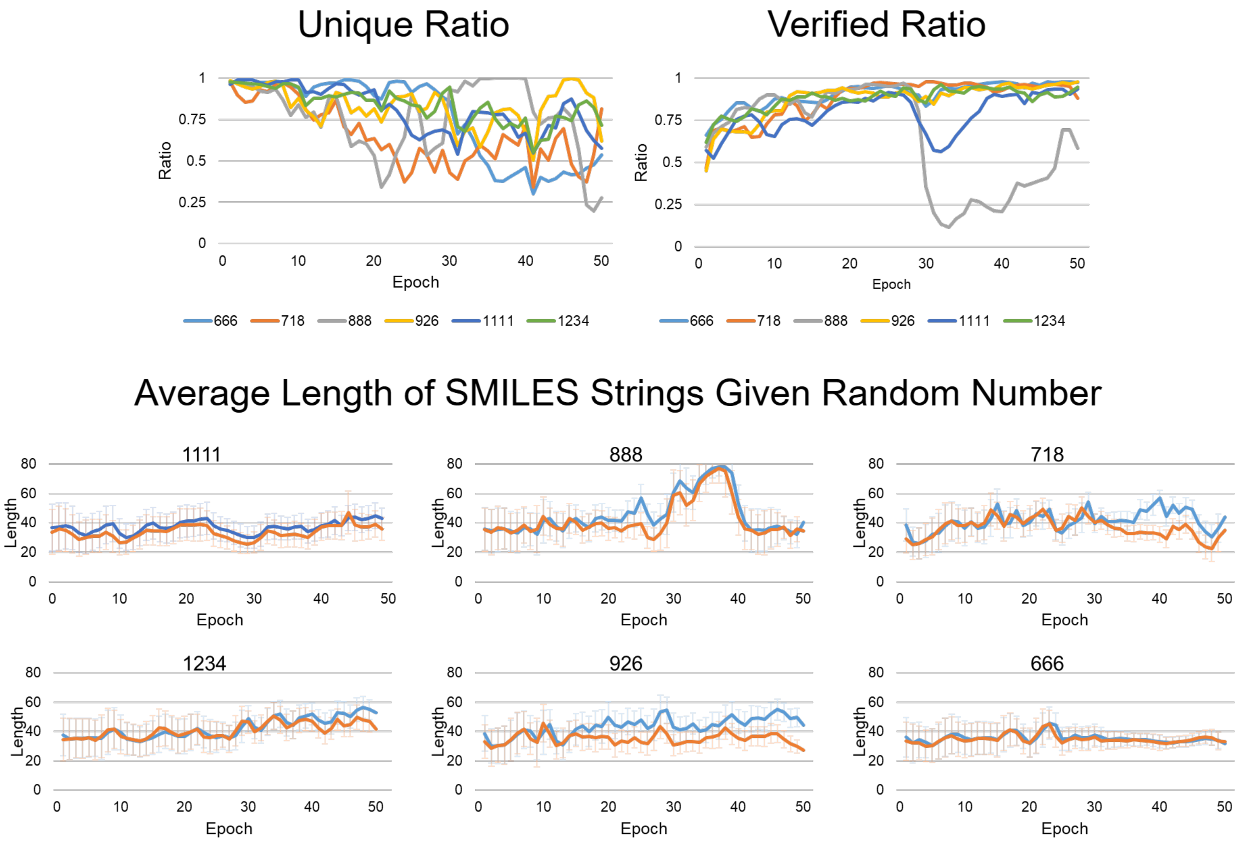

Figure S3: The impact of random number on the training process.

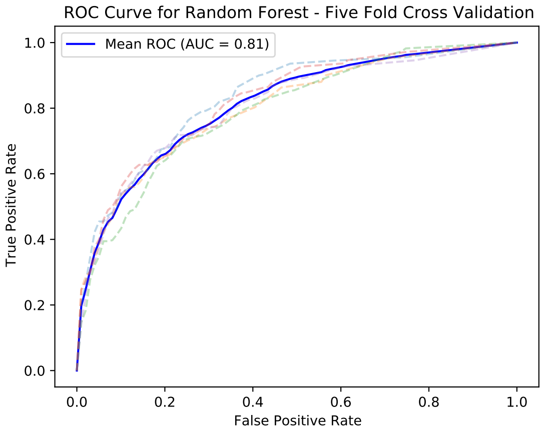

Figure S4: Evaluation of random forest classifier of CNS and non-CNS datasets

Features to be chosen as auxiliary input: 'TPSA', 'NumHDonors', 'NOcount', 'NumHeteroatoms', 'NumHAcceptors', 'VSA_EState3', 'SMR_VSA1', 'MinEStateIndex', 'PEOE_VSA1', 'Kappa3', 'ExactMolWt', 'Chi0'.

Parameters: n_estimators=100, random_state=42.

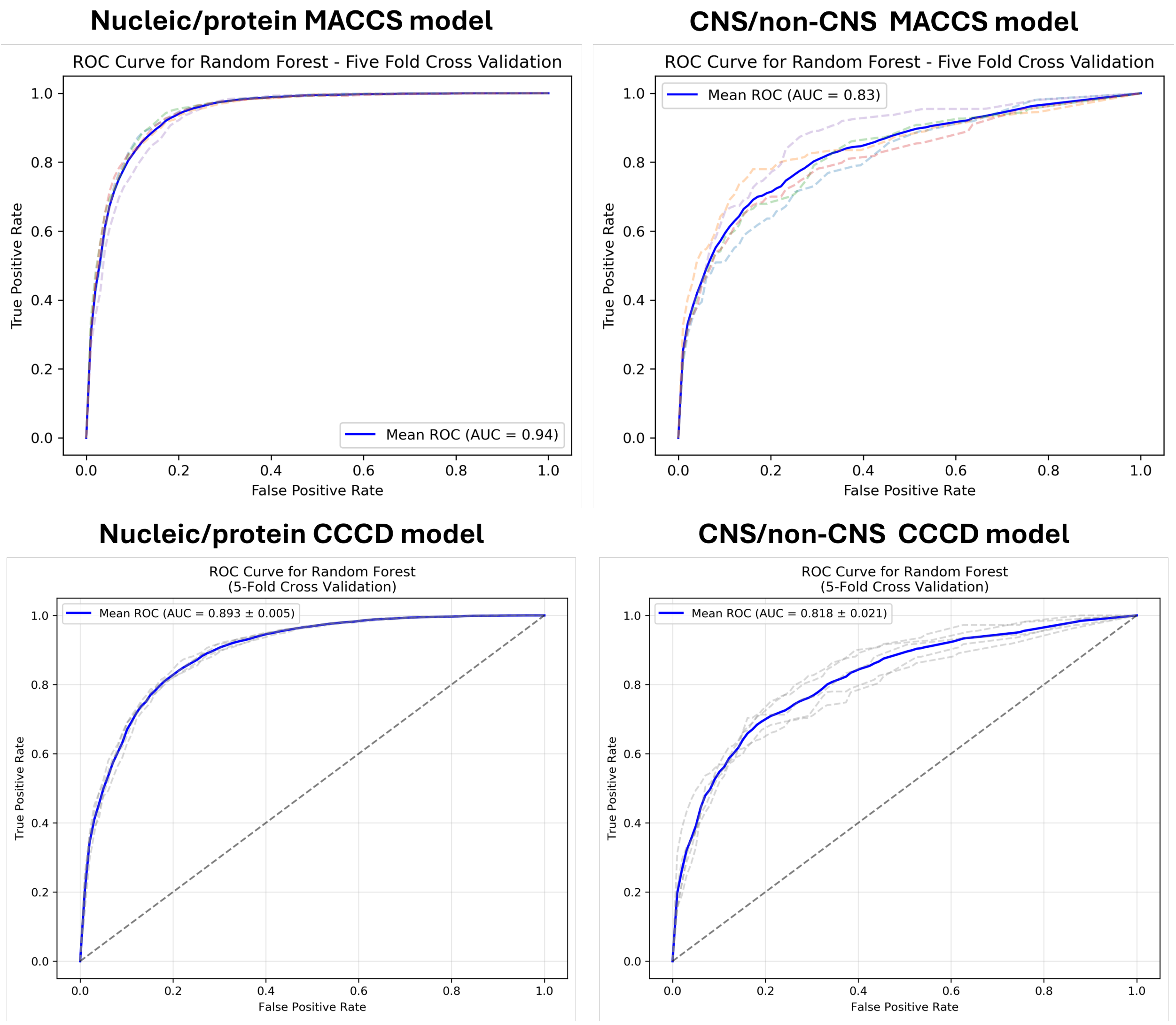

Figure S5: Evaluation of random forest classifier of datasets based on MACCS fingerprint and continuous and data-driven molecular descriptors.

For MACCS model, all the fingerprint are calculated by RDKit. For CDDD models, all the features are directly generated by CDDD. We then automatically chose the top 20 as descriptors for the random forest classifiers.

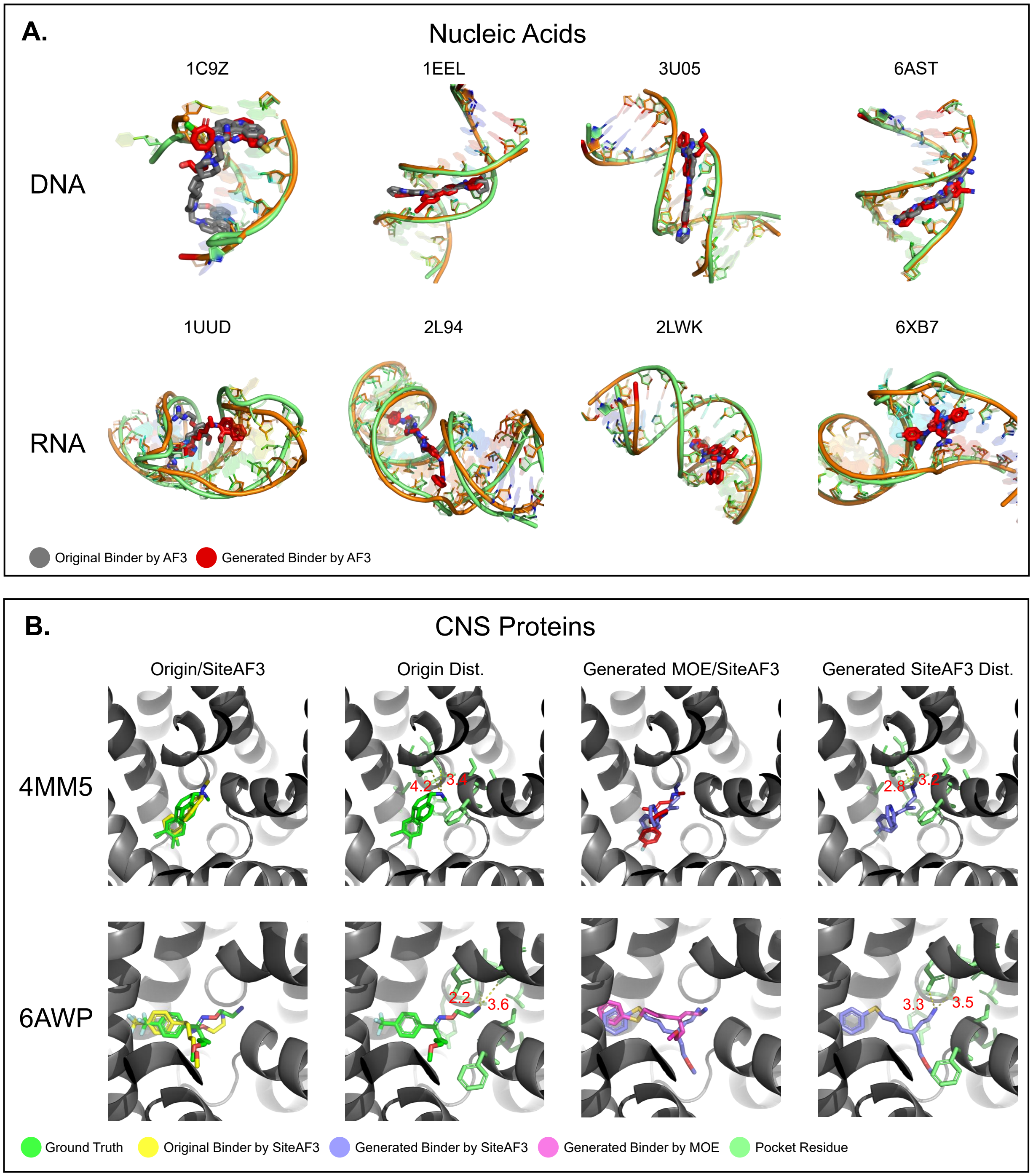

Figure S6: Binding pose evaluation of generated molecules with ground truth molecules by AlphaFold3 and SiteAF3. *A*. 8 cases of nucleic acid binders. *B*. Core interaction analysis of CNS-targeted small molecules.

Table S1: Binding pose evaluation of generated molecules with ground truth molecules by AlphaFold3 and SiteAF3.

Target Type	PDB ID	Original Binder (OB)	AF3 Ranking Score of OB	Generated Binder (GB)	AF3 Ranking Score of GB
DNA	1C9Z	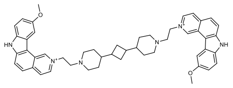	0.8317	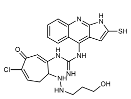	0.4862
	1EEL	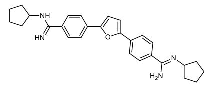	0.6299	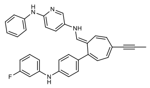	0.6037
	3U05	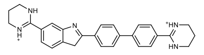	0.5890	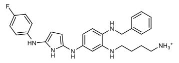	0.5969
	6AST	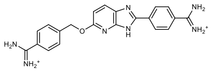	0.6334	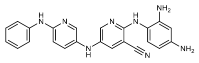	0.6308
RNA	1UUD	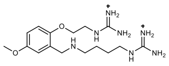	0.3888	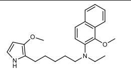	0.5078
	2L94	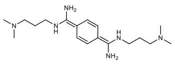	0.3979	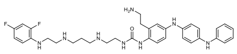	0.4421
	2LWK	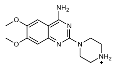	0.6840	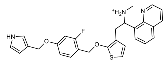	0.5966
	6XB7	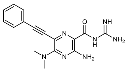	0.5775	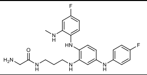	0.5262
CNS-protein	4MM5	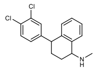	0.9479	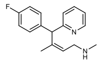	0.9265
	6AWP	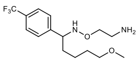	0.9647	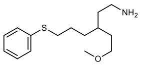	0.9543

Table S2: Docking results of selected targets and molecules.

Target Type	PDB ID	Original Binder (OB)	Docking Score of OB	Generated Binder (GB)	Docking Score of GB
DNA	1C9Z	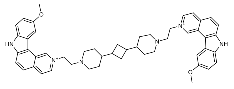	-155.153	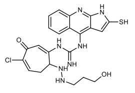	-110.624
				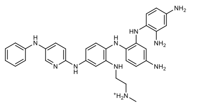	-101.655
	1EEL	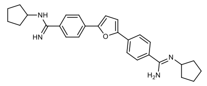	-112.678	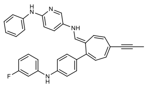	-124.438
				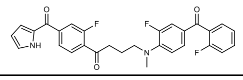	-117.033
	3U05	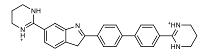	-140.703	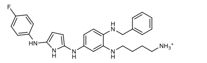	-135.616
				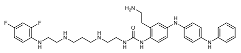	-132.885
	6AST	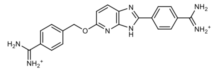	-111.071	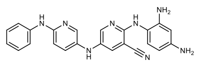	-111.136
				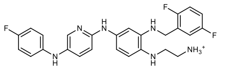	-112.929
RNA	1UUD	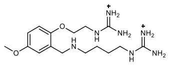	-54.498	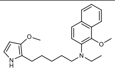	-54.738
				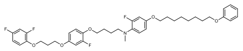	-72.612
	2L94	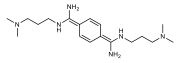	-58.012	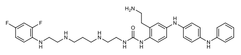	-95.692
				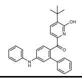	-60.546
	2LWK	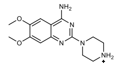	-50.534	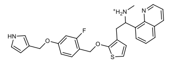	-52.455
				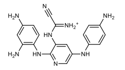	-61.176
	6XB7	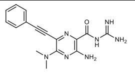	-69.094	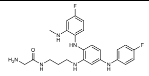	-79.227
				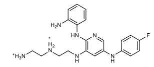	-80.270
CNS-protein	4MM5	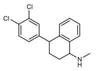	-6.4035	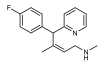	-6.7568
				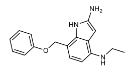	-7.2469
	6AWP	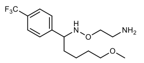	-6.2691	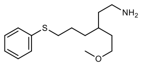	-6.6018
				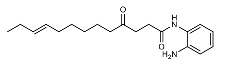	-7.9041

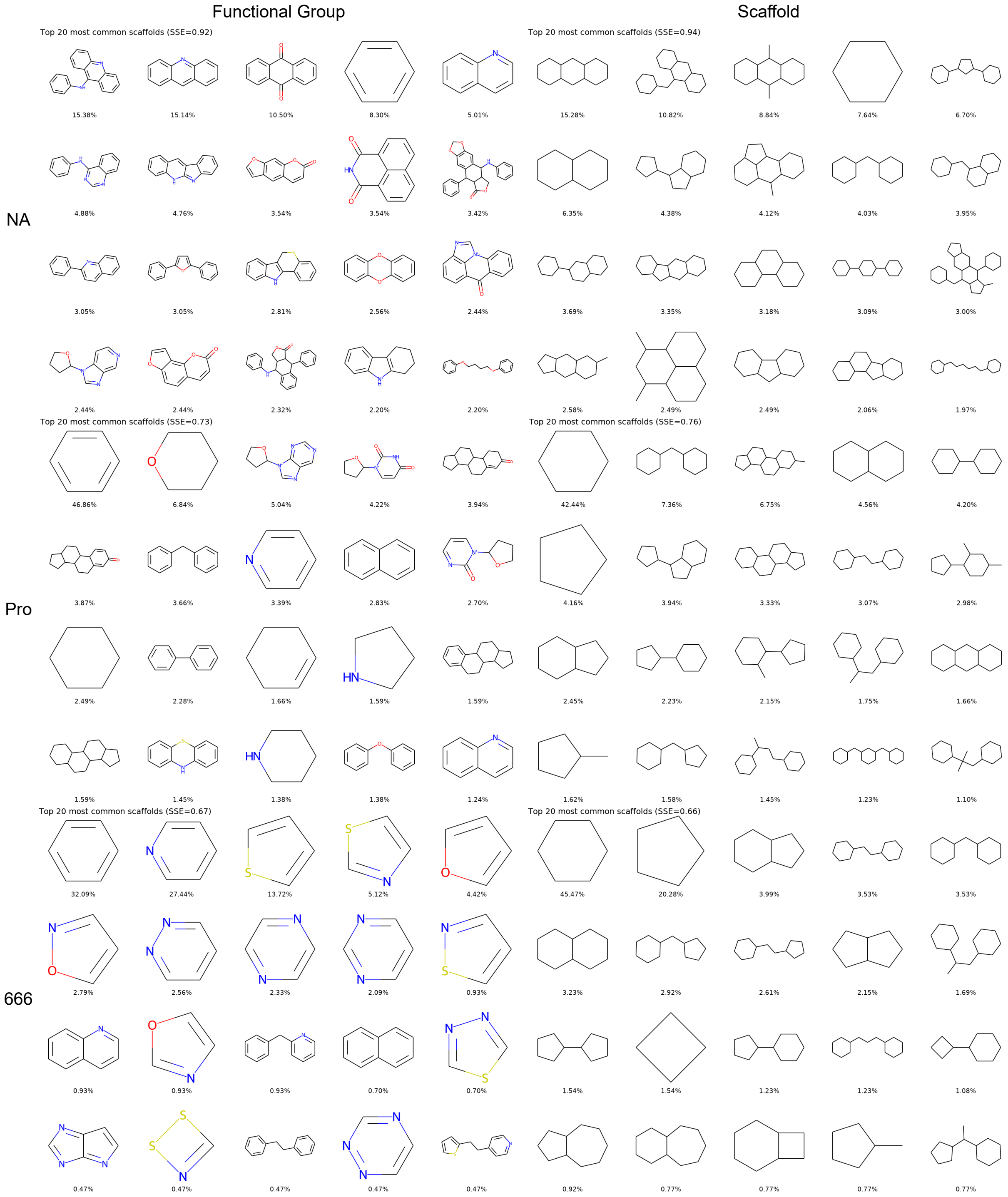
Functional Group
Scaffold
NA
Pro
666
Top 20 most common scaffolds (SSE=0.92)
15.38% 15.14% 10.50% 8.30% 5.01%
4.88% 4.76% 3.54% 3.54% 3.42%
3.05% 3.05% 2.81% 2.56% 2.44%
2.44% 2.44% 2.32% 2.20% 2.20%
Top 20 most common scaffolds (SSE=0.94)
15.28% 10.82% 8.84% 7.64% 6.70%
6.35% 4.38% 4.12% 4.03% 3.95%
3.69% 3.35% 3.18% 3.09% 3.00%
2.58% 2.49% 2.49% 2.06% 1.97%
Top 20 most common scaffolds (SSE=0.73)
46.86% 6.84% 5.04% 4.22% 3.94%
3.87% 3.66% 3.39% 2.83% 2.70%
2.49% 2.28% 1.66% 1.59% 1.59%
1.59% 1.45% 1.38% 1.38% 1.24%
Top 20 most common scaffolds (SSE=0.76)
42.44% 7.36% 6.75% 4.56% 4.20%
4.16% 3.94% 3.33% 3.07% 2.98%
2.45% 2.23% 2.15% 1.75% 1.66%
1.62% 1.58% 1.45% 1.23% 1.10%
Top 20 most common scaffolds (SSE=0.67)
32.09% 27.44% 13.72% 5.12% 4.42%
2.79% 2.56% 2.33% 2.09% 0.93%
0.93% 0.93% 0.93% 0.70% 0.70%
0.47% 0.47% 0.47% 0.47% 0.47%
Top 20 most common scaffolds (SSE=0.66)
45.47% 20.28% 3.99% 3.53% 3.53%
3.23% 2.92% 2.61% 2.15% 1.69%
1.54% 1.54% 1.23% 1.23% 1.08%
0.92% 0.77% 0.77% 0.77% 0.77%

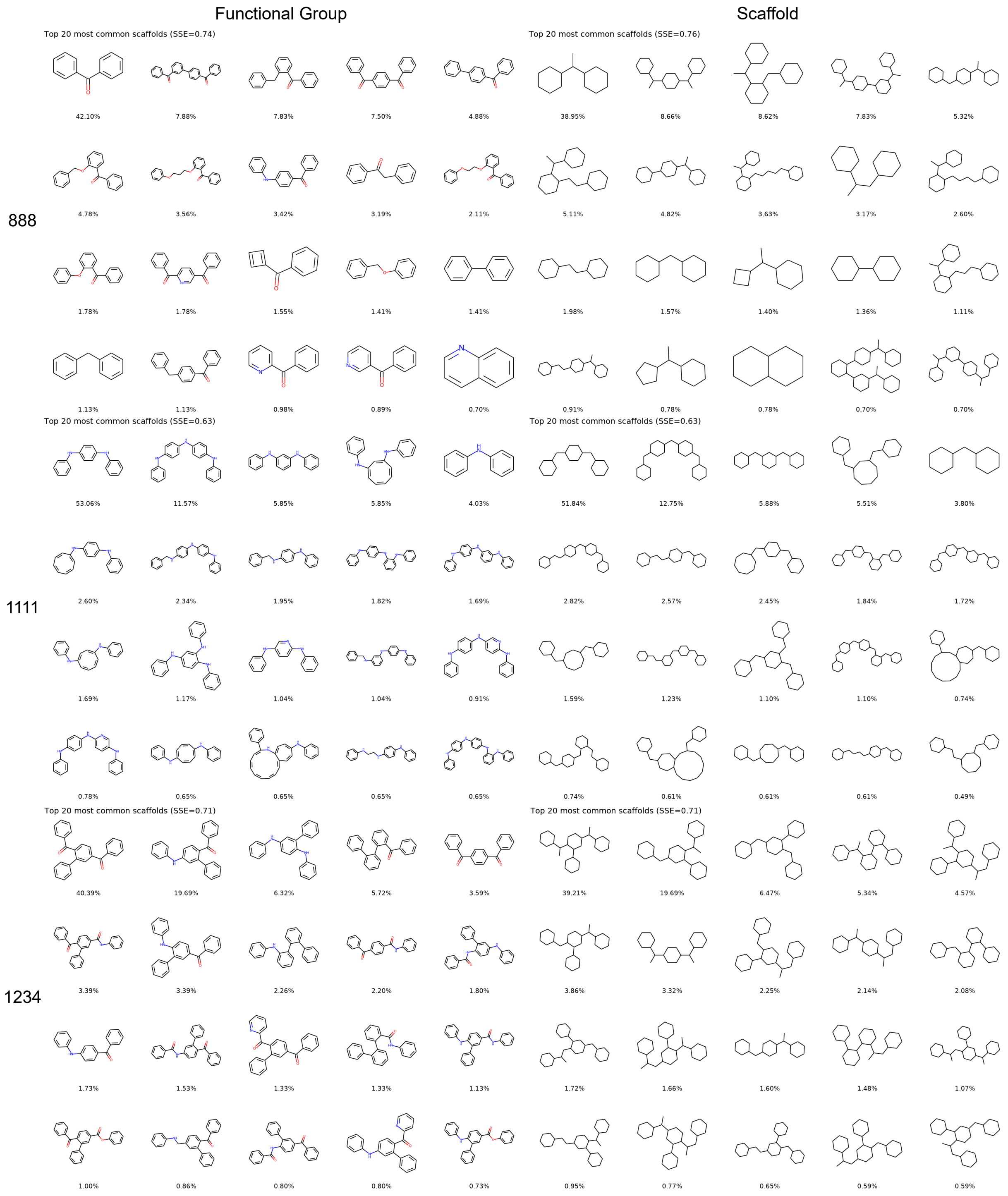

Figure S7: Functional group and scaffold analysis of ground truth of nucleic acid and protein binders and generated molecules according to different random number.

CB1 case study

Construction of LRIP-SF scoring function for CB1R.

We have collected 2802 CB1R compounds with measured Ki values from ChEMBL database.[1] Ligand-receptor interaction-profile scoring function (LRIP-SF) was constructed for the 2251 randomly selected compounds. Details on how to construct LRIP-SF were described in our previous publication.[2,3] Exceptions departure from the established protocol include using ABCG2[4] instead of AM1-BCC charge model for the ligands, LRIP-SFs were constructed using all the 28 machine learning algorithms implemented in Matlab R2024a's Regression-Learner module. Rational quadratic Gaussian Processing Regression achieved the best performance with an RMSE of 1.30 kcal/mol in the ten-fold cross validation. The model was then applied to make predictions for the 551 testing compounds and achieved an RMSE of 1.27 kcal/mol and R2 of 0.411, significantly outperforming Glide docking, for which the RMSE and R2 are 2.14 kcal/mol and 0.0001, respectively.

A binary classification model for CB1 binders.

As requested by the ADSeqGAN architecture, a classification model is required to generate auxiliary loss. Unlike the cases of nucleic acid binders and CNS drugs, we applied MACCS fingerprint to construct the classification model. We grouped the CB1 ligands into two groups, the compounds which have ki values better than 1µM belonging to the active group while the rest of the compounds belonging to inactive group. The model performance is shown in figure S8A.

Evaluation of ADSeqGAN-generated molecules.

We preprocessed the ADSeqGAN designed 17157 valid molecules (total 32000) using the following filters: the calculated QED drug likeness score is no worse than 0.6, molecular weight is no smaller than 150 and no larger than 500, the numbers of hydrogen bond acceptors

and donors are no more than 10 and 5, respectively, the calculated logP value is no larger than 5.0. After applying those filters, the remaining 6059 druglike molecules were subjected to Glide docking screening followed by the binding affinity calculation using the established LRIP-SF model.

We then grouped those druglike molecules into the active and inactive groups based on their LRIP-SF scores. If a threshold of -8.1854 kcal/mol, which corresponds to 1 µM, was used to determine if a compound is active or inactive, 32.8% of designed molecules belong to the active group, while when a more stringent threshold, -9.0 kcal/mol, corresponding to 0.25 µM, was applied, still 6% of the designed compounds belong to the active group. Overall, the molecular set of the designed molecules is of high quality containing a higher percentage of CB1R ligands than the general-purpose screening libraries and the focused screening libraries for CB1R. The distribution of the LRIP-SF scores for the designed molecules is shown in Figure S8D. The one with the best predicted LRIP-SF score of -12.10 kcal/mol, corresponding to 1.5 nM, is shown as green sticks in Figure S8B. The molecule forms two hydrogen bonds with Ser 383, a key residue for ligand binding at the orthostatic binding site of CB1R.[5,6]

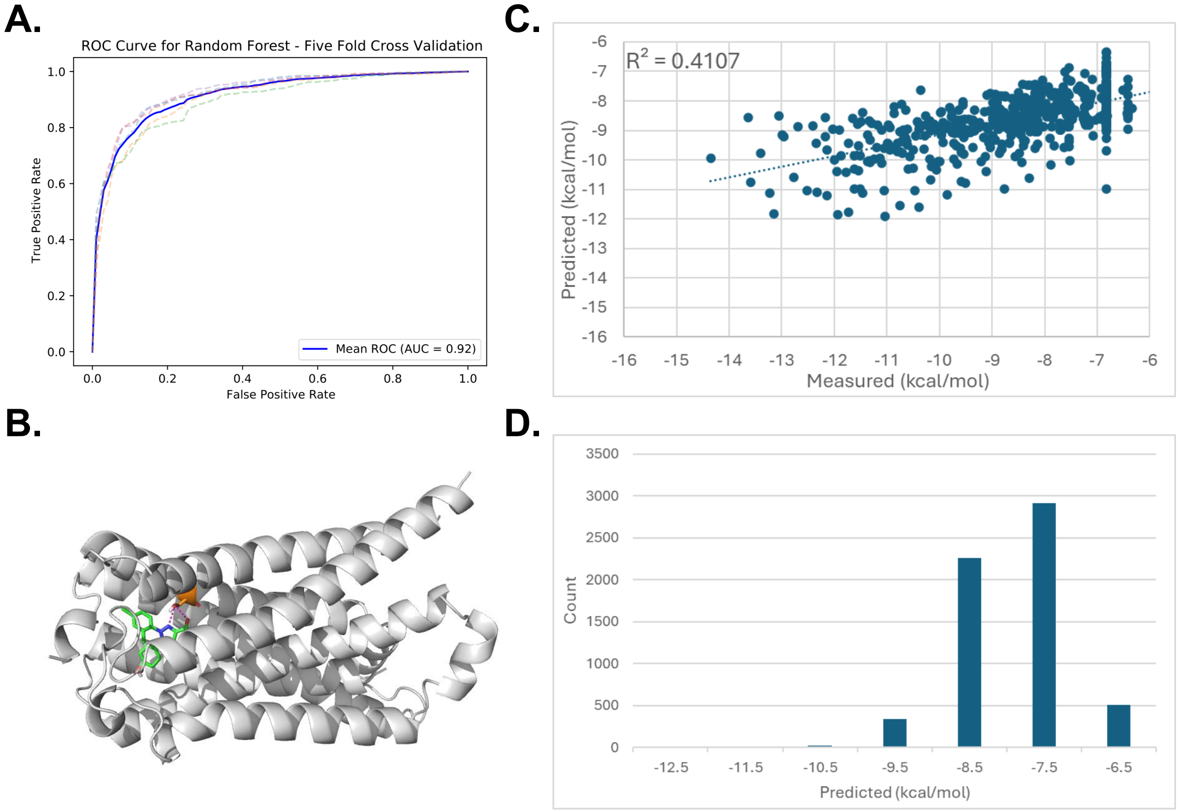

Figure S8: Performance of ADSeqGAN for CB1R drug target. *A*. MACCS fingerprint based random forest classifier of CB1 active and non-active ligands. *B*. Top 1 ADSeqGAN-designed CB1R molecule (green sticks) binding to CB1R (grey cartoon) with two hydrogen bonds (dashed magenta lines) formed with Ser 383 (brown sticks). *C*. Performance of LRIP-SF model for the test set compounds. *D*. Distribution of the predicted LRIP-SF scores for the designed druglike molecules.

Computational Details

Computation can be down either on CPU and GPU.

For nucleic acid and protein dataset, training can be down in 20 hours using 8 cores on AMD EPYC 9374F. For central nervous system drugs, training can be down in approximately 15 hours using 8 cores on AMD EPYC 9374F. For CB1 drugs, training can be down in approximately 13 hours using 8 cores on AMD EPYC 9374F. For generation, it only takes several seconds to generate more than 10 thousands molecules on AMD EPYC 9374F.

We also provide GPU version code online.

All the training parameters can be found in **condi_example.py** for NA training and **condi_cns.py** for CNS training on https://github.com/HaCTang/ADSeqGAN.

References

(1) Mendez, D.; Gaulton, A.; Bento, A. P.; Chambers, J.; De Veij, M.; Félix, E.; Magariños, M. P.; Mosquera, J. F.; Mutowo, P.; Nowotka, M.; others ChEMBL: towards direct deposition of bioassay data. *Nucleic acids research* **2019**, *47*, D930–D940.

(2) Ji, B.; He, X.; Zhai, J.; Zhang, Y.; Man, V. H.; Wang, J. Machine learning on ligand-residue interaction profiles to significantly improve binding affinity prediction. *Briefings in Bioinformatics* **2021**, *22*, bbab054.

(3) Niu, T.; Wang, N. X.; Wang, J. *Machine learning and deep learning based scoring functions in deciphering ligand-receptor binding: An application in drug design for GPCRs*; Elsevier, 2024; pp 189–224, [Online; accessed 2025-09-06].

(4) He, X.; Man, V. H.; Yang, W.; Lee, T.-S.; Wang, J. ABCG2: A Milestone Charge Model for Accurate Solvation Free Energy Calculation. *Journal of Chemical Theory and Computation* **2025**, *21*, 3032–3043, [Online; accessed 2025-09-06].

(5) Murrieta, A. C.; Mendoza-Espinosa, P.; Velasco-Bolom, J. L.; Contreras-Torres, F. F. Identification and structural characterization of CB1 receptor antagonists: A comprehensive virtual screening and molecular dynamics study of arachidin-2. *Biophysical Chemistry* **2025**, *318*, 107385, [Online; accessed 2025-09-06].

(6) Haider, S.; Pandey, P.; Reddy, C. R.; Lambert, J. A.; Chittiboyina, A. G. Novel Machaeriol Analogues as Modulators of Cannabinoid Receptors: Structure–Activity Relationships of (+)-Hexahydrocannabinoids and Their Isoform Selectivities. *ACS omega* **2021**, *6*, 20408–20421.
